# Simplifying Sample Preparation for Soil Fertility Analysis by X-ray Fluorescence Spectrometry †

**DOI:** 10.3390/s19235066

**Published:** 2019-11-20

**Authors:** Tiago Rodrigues Tavares, Lidiane Cristina Nunes, Elton Eduardo Novais Alves, Eduardo de Almeida, Leonardo Felipe Maldaner, Francisco José Krug, Hudson Wallace Pereira de Carvalho, José Paulo Molin

**Affiliations:** 1 Laboratory of Precision Agriculture (LAP), Department of Biosystems Engineering, “Luiz de Queiroz” College of Agriculture (ESALQ), University of São Paulo (USP), Piracicaba, São Paulo 13418900, Brazil; tiagosrt@usp.br (T.R.T.); leonardofm@usp.br (L.F.M.); 2 Laboratory of Analytical Chemistry (LQA), Center for Nuclear Energy in Agriculture (CENA), University of São Paulo (USP), Piracicaba, São Paulo 13416000, Brazil; lcnunes@cena.usp.br (L.C.N.); fjkrug@cena.usp.br (F.J.K.); 3 Laboratory of 14 Carbon (LC14), Center for Nuclear Energy in Agriculture (CENA), University of São Paulo (USP), Piracicaba, São Paulo 13416000, Brazil; elton.alves@usp.br; 4 Laboratory of Nuclear Instrumentation (LIN), Center for Nuclear Energy in Agriculture (CENA), University of São Paulo (USP), Piracicaba, São Paulo 13416000, Brazil; edualm@cena.usp.br (E.d.A.); hudson@cena.usp.br (H.W.P.d.C.)

**Keywords:** precision agriculture, X-ray fluorescence, spectroscopy, soil nutrients, proximal soil sensing, soil testing

## Abstract

Portable X-ray fluorescence (pXRF) sensors allow one to collect digital data in a practical and environmentally friendly way, as a complementary method to traditional laboratory analyses. This work aimed to assess the performance of a pXRF sensor to predict exchangeable nutrients in soil samples by using two contrasting strategies of sample preparation: pressed pellets and loose powder (<2 mm). Pellets were prepared using soil and a cellulose binder at 10% w w^−1^ followed by grinding for 20 min. Sample homogeneity was probed by X-ray fluorescence microanalysis. Exchangeable nutrients were assessed by pXRF furnished with a Rh X-ray tube and silicon drift detector. The calibration models were obtained using 58 soil samples and leave-one-out cross-validation. The predictive capabilities of the models were appropriate for both exchangeable K (ex-K) and Ca (ex-Ca) determinations with R^2^ ≥ 0.76 and RPIQ > 2.5. Although XRF analysis of pressed pellets allowed a slight gain in performance over loose powder samples for the prediction of ex-K and ex-Ca, satisfactory performances were also obtained with loose powders, which require minimal sample preparation. The prediction models with local samples showed promising results and encourage more detailed investigations for the application of pXRF in tropical soils.

## 1. Introduction

Brazil is the fourth largest consumer of fertilizers in the world [1] due to the predominance of acidic and low fertility tropical soils. Thus, the diagnosis of soil fertility is crucial for the correct management of fertilizers and limestone in crops. Per year, it is estimated that about 1 million soil tests are carried out by Brazilian fertility analysis laboratories. There is an expectation of increasing this number, due to the expansion of agricultural areas [2], as well as the adoption of soil mapping techniques for precision agriculture (PA) practices, which demand information in a high spatial and temporal density [3]. In addition, as commented by Demattê et al. [2], traditional soil analyses face other challenges related to the time required for performing the laboratory measurements (about 3 to 15 days), and also the hazardous reagents still used in some tests (e.g., dichromate and sulfuric acid).

The establishment of a robust method for the direct analysis of soils using sensing techniques—allowing farmers and laboratories to increase the number of analyses in a practical and clean way, without relying exclusively on traditional fertility soil tests—is a current need in Brazil and all over the world [3]. This task is a great multidisciplinary challenge for the researchers involved [4]. Hence, discussions have recently begun between academics [2] and companies for the development of hybrid laboratories. The term hybrid refers to labs, where analyses performed by sensor systems are used in combination with the traditional methods, allowing one to use the sensing techniques to predict some soil attributes. Hybrid laboratories are compatible with controlled-environment and on-field analyses (e.g., using a mobile soil-testing lab [5]). This is an interesting strategy, which should boost worldwide research in the coming years to seek the best set of sensors compatible with direct analysis of soils, as well as the best strategy for calibration of the predictive models.

X-ray fluorescence (XRF) is a spectroanalytical technique compatible with direct soil analysis, which can be applied with minimal or no sample preparation [6]. The recent technological advance of the optical and electronic components allowed the development and miniaturization of this technology, and it has become attractive for use in hybrid laboratories and in situ analyses. Some studies have already pointed out the potential of using XRF sensors in proximal soil sensing (PSS) approaches [7,8]. Despite that, XRF has been poorly explored for assessments of physical and chemical attributes of tropical soils, mainly under the context of PSS and PA.

To use XRF sensors as a practical analytical method in hybrid laboratories—in order to ensure a massive increase in the number of samples analyzed—it should be compatible with a simple soil sample preparation (e.g., just air-dried and sieved rapidly). Recent studies involving XRF sensors for practical analysis of soil attributes have used dried samples with particle sizes smaller than 2 mm [8,9,10,11]. It is a consensus that pellet preparation after grinding the soil allows one to explore the potential of the XRF technique in soil analysis [12]. The preparation of a pellet is recommended for analyses with the XRF technique because it improves the homogeneity of the material and also allows one to control the density, porosity and surface roughness characteristics, reducing the physical matrix effects [13]. Although it is known that the preparation of pellets guarantees better precision in the measurements performed with the XRF [14,15], recent studies have assumed that, when analyzing soil samples with particle sizes smaller than 2 mm, its heterogeneity and physical matrix effects can be neglected.

XRF analyses are more flexible with regards to sample preparation because—unlike other elemental analysis techniques, such as laser induced breakdown spectroscopy (LIBS)—they also allow one to evaluate loose powder [14]. However, we did not find any study comparing XRF performance for the prediction of fertility attributes on soil samples that were just dried and sieved (<2 mm) with samples prepared with the optimal sample preparation method. Therefore, the level of performance loss when neglecting the physical matrix effect and heterogeneity is unknown. For a robust development of the XRF technique as a practical tool for soil fertility analysis, one of the key points is to understand the tradeoff between analytical performance and sample preparation, in order to reduce or eliminate these procedures based on the analytical potential of the sensor for each sample condition. Such knowledge is important for the development of PSS applications using this tool.

To evaluate the possibility of simplifying the sample preparation procedure for XRF analyses, this work aimed to assess the performance of a portable XRF (pXRF) to predict exchangeable nutrients in soil samples prepared using two contrasting types of sample preparation: pellets and loose powder (≤2 mm). The effect of sample preparation in the spatial distribution of nutrients on the sample surface was evaluated using a benchtop microprobe X-ray fluorescence spectrometry (µ-XRF). Moreover, a procedure for preparation of soil pellets, involving planetary ball milling and the use of binding agents was also assessed.

## 2. Material and Methods

### 2.1. Soil Samples

A set of 58 soil samples were selected for the comparison of their exchangeable nutrients content with the X-ray fluorescence produced by the pellet and loose powder samples. These samples were collected from 0 to 0.2 m soil depth in an agricultural field located at the southeast region of Brazil, in the municipality of Piracicaba, state of São Paulo (at coordinates 22°41′57.24″ S and 47°38′33.33″ W, WGS84 datum). The soil is classified as Lixisol [16] with a clayey texture and high nutrient variability. 

The soil samples were air-dried and sieved (<2 mm) and after that, three subsamples were separated: (i) 0.8 g was used for pelletizing, (ii) 10 g was analysed as loose powder, and (iii) about 30 g was used for the reference measurements.

### 2.2. Sample Preparation

**Figure 1:**
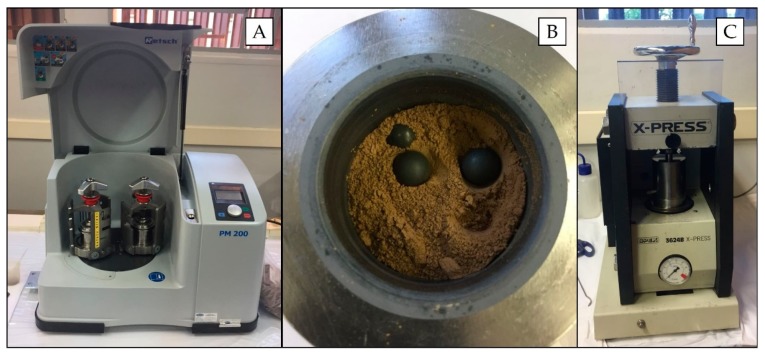
Planetary ball mill (**A**), loose soil inside the tungsten carbide jars with the tungsten carbide balls (**B**) and hydraulic press (**C**), which were used at work.

For pelletizing, the samples (particles < 2 mm) were initially dried at 105 °C for 24 h and thereafter ground in a planetary ball mill (Retsch model PM 200 mill, Germany) (Figure 1A) by using two grinding tungsten carbide jars (50 mL; Retsch, Germany) with 10 tungsten carbide balls (10 mm diameter) (Figure 1B). Grinding was performed at 400 rpm for 5 min clockwise/5 min counter clockwise with a 10-s stop before changing the rotation direction.

**Figure 2:**
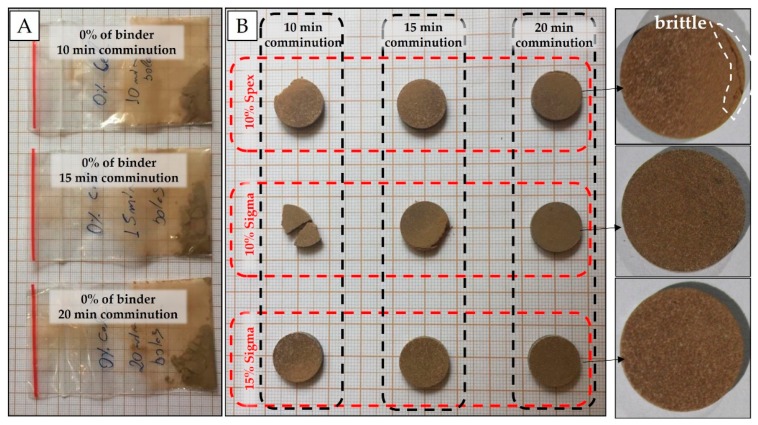
Soil samples without the addition of binder and with different grinding times (**A**); pellets resulting from tests with different grinding times, cellulose concentrations and brands (**B**).

Preliminary experiments were carried out by just pressing the soil samples without a binder. It was observed that for the sandier sample (clay content of 175 g dm^−3^) the resulting pellets were friable and easily crumbled (Figure 2A). Therefore, binder addition was decisive for improving the quality of the pellets. The binders tested were chosen based on their similarity to the analytical blank, i.e., lower analyte mass fractions of elements evaluated in the soil fertility (e.g., P, K, Ca, and Mg). In this case, binding agents, such as a microcrystalline cellulose powder (Sigma-Aldrich, Merck, Darmstadt, Germany), and cellulose (SPEX 3642, Metuchen, NJ, USA) were evaluated in the proportion of 10 and 15% w w^−1^, with grinding/homogenization times of 10, 15, and 20 min.

The grinding and homogenized samples were pelletized in a hydraulic press (SPEX 3624B X-Press) (Figure 1C) by transferring 0.8 g of the powdered material to a stainless steel set and applying 8.0 t cm^−2^ for 3 min. Cylindrical pellets were approximately 15 mm diameter and 2 mm thick, with mass per unit area of 0.45 g cm^−2^. The pellets were visually inspected, evaluating their homogeneity aspect and integrity. Furthermore, an XRF spectra (obtained with pXRF, as described in Section 2.5) of a pellet and a loose soil sample were also compared in order to assess possible contamination during the milling process. Further experiments were performed with 10% w w^−1^ cellulose binder (Sigma-Aldrich, Merck, Darmstadt, Germany) and 20 min of grinding in a planetary ball mill.

Sample presentation in the form of loose powder was also considered for the analysis. The air-dried samples were sieved in a sieve with apertures of 2 mm. Ten grams of test sample was transferred to an XRF polyethylene cup of 31 mm (n. 1530, Chemplex Industries Inc., Palm City, FL, USA) assembled with a 4-μm thick polypropylene film (n. 3520, SPEX, USA).

### 2.3. Soil Laboratory Analysis

Soil testing conducted by a commercial laboratory determined the exchangeable (ex-) contents of P, K, Ca and Mg via ion exchange resin extraction. Clay content was quantified by the Bouyoucos hydrometer method in dispersing solution. The pseudo total content (ptc) of P, K, Ca and Mg were also analyzed following the USEPA Method 3051A [17]. The latter methods involve the extraction of ions using HNO_3_ and HCl. The multielement quantification was made by inductively-coupled plasma optical emission spectrometry (ICP OES). The term ptc is used, because it is not a total digestion method. Despite this, this method presents proportional recoveries to the most aggressive methods for the determination of elements in tropical soils [18], allowing one to understand the relationship between exchangeable and total content of the elements evaluated. In addition, it is a method that requires less time for digestion, less consumption of acids and lower risks of environmental contamination [19].

### 2.4. μ-XRF Chemical Images

µ-XRF is a type of energy dispersive X-ray fluorescence that employs a micrometric beam with a shape and size defined by a primary optic element; this can be done by a simple collimator, an optical capillary or a focusing mirror [20,21]. In this work, the µ-XRF technique was employed to characterize, on the sample surface, the influence of the sample preparation method on the spatial distribution of elements of interest.

The net intensities for K and Ca Kα emission lines were characterized with high spatial resolution on the surface of loose soil and pellet samples. A benchtop µ-XRF system (Orbis PC EDAX, United States) furnished with a Rh anode X-ray tube was used. The detection was carried out by a 30 mm^2^ silicon drift detector (SDD). The µ-XRF tube current and voltage was operated at 15 kV and 200 μA, respectively; the beam size used was 30 μm and no primary filter was used; the live time was set to 2 s per spot; and the analysis was carried out under vacuum. In each sample, 800 points (matrix of 32 × 25 points) were evaluated in an area of about 2.32 mm^2^ (1.60 × 1.45 mm).

Chemical images showing the variability of K and Ca, produced by Orbis Vision software, were linearly interpolated using Origin Lab 2016. The mean, maximum and minimum values, as well as the coefficient of variation (CV)—the ratio between the standard deviation and the mean expressed in percentage—were also calculated. P and Mg Kα emission lines were not identified in the samples, which did not allow the evaluation of these element lines. Similar µ-XRF analysis procedures are described by Rodrigues et al. [21].

### 2.5. pXRF Measurements and Its Performance Evaluation

The measurements were carried out using a portable X-ray fluorescence spectrometer (portable ED-XRF), Tracer III–SD model (Bruker AXS, Madison, USA), equipped with a 4 W Rh X-ray tube and 12 mm^2^ of active area, and a X-Flash® Peltier-cooled SDD, with 2048 channels (Bruker AXS, Madison, USA). The tube operated at 23 μA and 15 kV, and emission intensities were measured for 90 s without vacuum. The voltage configuration was chosen based on the interest in low atomic number elements and the current, in order to keep deadtime below 15%, and avoiding spectral distortions and artifacts. Soil samples were measured in triplicate at different portions of its surface. To ensure the same attenuation conditions of the loose soil samples, the pellets were placed on a 4-μm-thick polypropylene thin-film.

All data were acquired using the software Bruker S1PXRF® (Bruker AXS, Madison, USA). The data were obtained through the deconvolution process using the Artax® (Bruker AXS, Madison, USA). The Kα emission characteristic lines of the elements P, K, Ca and Mg were evaluated. However, only K and Ca presented detectable emission lines, which were evaluated by their signal-to-noise ratio (SNR) and intensity, through the counts of photons per second (cps). The SNR was determined by dividing the characteristic X-ray net intensities by the background square root [22], and the cps were obtained by the ratio of total X-ray intensity to detector live time. The standard deviation (SD) behavior of the intensity of the K and Ca Kα emission lines within the replicates was also evaluated for both sample preparations.

The intensity of K and Ca Kα emission lines, obtained from pellets and loose soil samples, were compared with the exchangeable contents of these nutrients. Calibration models were built using simple linear regressions (a univariate model), with the independent variable being pXRF data and the dependent variable being the soil property measured via commercial laboratory procedures. The prediction models were validated using “leave-one-out” full cross-validation. The quality of the developed calibration was assessed with the coefficient of determination (R^2^), the root mean square error (RMSE) and the ratio of performance to interquartile range (RPIQ), as recommended by Bellon-Maurel et al. [23]. Arbitrary groups were used for simplification of interpretation, as proposed by Nawar and Mouazen [24]: (1) excellent models (RPIQ > 2.5), (2) very good models (2.5 > RPIQ > 2.0), (3) good model (2.0 > RPIQ > 1.7), fair (1.7 > RPIQ > 1.4), and very poor model (RPIQ < 1.4). The descriptive statistics of soil fertility and the correlation between pseudo total and exchangeable contents were also determined.

## 3. Results

### 3.1. Soil Pelletizing Procedure

The pelletizing of sandy soil samples (e.g., about 175 g dm^−3^ of clay content) was only possible with the addition of binder. In the initial tests, which evaluated different grinding times, they did not form pellets without the addition of binder (Figure 2A). In general, pellets produced after 10 and 15 min of grinding were brittle, except for pellets containing 15% w w^−1^ of binder (Figure 2B), which, in turn, were less homogeneous with white spots on their surface. The best cohesion between particles was obtained with 20 min of grinding. For this milling time, the pellet with 15% w w^−1^ of binder was slightly less heterogeneous than the pellet with 10% w w^−1^. In relation to the brand, microcrystalline cellulose powder (Sigma-Aldrich, Merck, Darmstadt, Germany) presented more cohesive pellets. The best results were observed for pressed pellets prepared from soil mixed with cellulose binder at 10% w w^−1^ and ground for 20 min.

**Figure 3:**
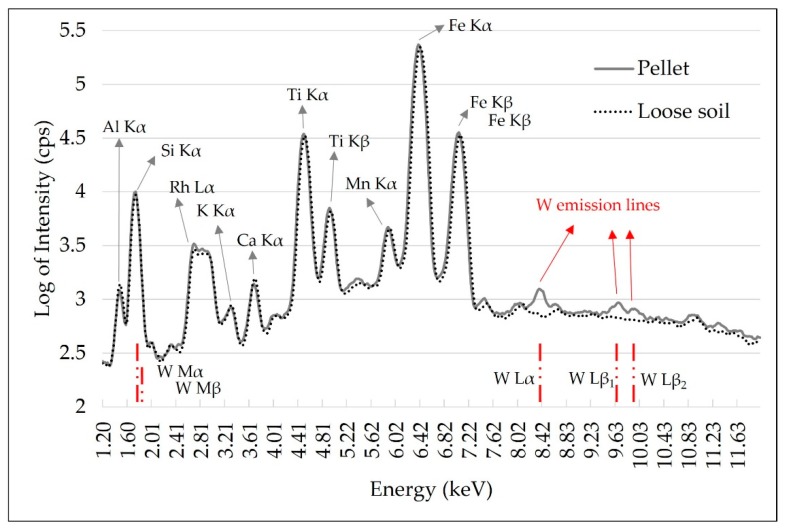
X-ray fluorescence spectra obtained with pXRF equipment using the pellet and loose soil sample. Tungsten (W) emission lines were identified with red lines. The emission spectra intensity is shown in the logarithm to reduce the differences in scales between the emission lines, allowing a better qualitative assessment.

Tungsten (W) contamination was observed in these ground soil samples. This contamination was caused by the tungsten carbide ball mill and it was evidenced by the W L-emission lines presented in the pellet spectrum, which were not observed in the loose soil spectrum (Figure 3). In the XRF spectra, W presents L and M-emission lines with energy lower than 15 keV, as highlighted in the red lines in the spectrum of Figure 3. In this range, the main W emission lines present energy of 1.77 (Mα), 1.83 (Mβ), 8.39 (Lα), 9.67 (Lβ_1_) and 9.95 keV (Lβ_2_). The effect of this contamination is best seen on L-emission lines, as they do not overlap with any other emission lines present. The W M-α line overlaps with Si (1.74 keV) and can also promote the enhancement of the Al Kα line (Al K edge = 1.55 keV) due to secondary radiation excitation (chemical matrix effect). Thus, this W interference must be considered and corrected in the case of Al quantification. For the Ca and K determinations performed in this work, contamination with W was not a limiting factor.

### 3.2. μ-XRF Chemical Images and Sample Homogeneity

**Figure 4:**
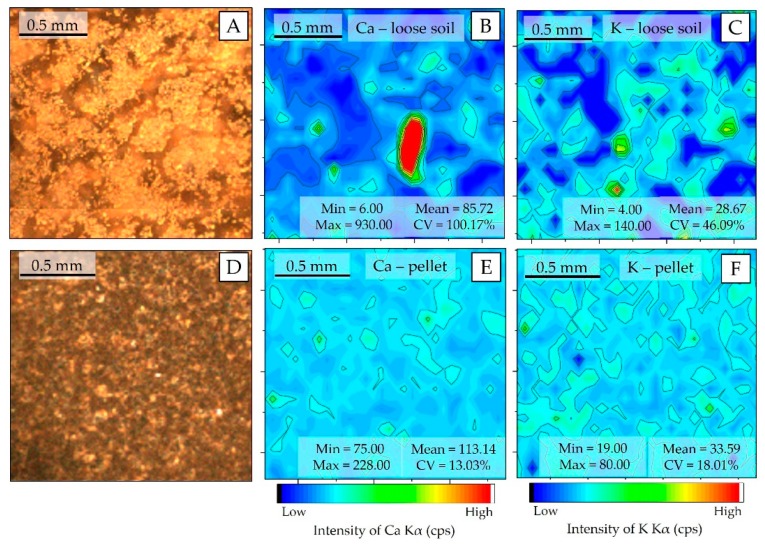
μ-XRF chemical images showing the spatial distribution of Ca and K in the loose soil (**B** and **C**, respectively); Ca and K in the pellet (**E** and **F**, respectively). Photo of the analyzed area of (**A**); the loose soil (**A**) and pellet (**D**).

The spatial distribution patterns of Ca and K at the pellet and loose soil surface are shown in Figure 4. For both elements, the preparation of pellets resulted in more homogeneous surfaces than those observed for loose soil. The particle size reduction—required for pellet production—allows one to homogenize the distribution of the different elements in the sample, which occurs because it fragments the regions where these elements are agglomerated (nuggets). In the loose soil sample, the presence of nuggets can be observed for the Ca and K (Figure 4B,C, respectively), which do not appear in the pelletized samples. The homogenization promoted by pelletizing drastically reduced the CV of both Kα emission line intensities, oscillating from 100.17% to 13.03% for Ca and from 46.09% to 18.01% for K, respectively.

### 3.3. Soil Exchangeable Nutrient Prediction Using a pXRF Spectrometer

Soil samples were characterized by clayey texture, low variability of clay content (between 345 and 511 g dm^−3^) and high variability of exchangeable nutrients. According to the local fertility interpretation [25], the level of ex-P content oscillates between low to medium; and the level of ex-K, ex-Ca and ex-Mg content varies between medium to very high. These samples are also characterized by a significant correlation between available and pseudo total contents for all nutrients. A descriptive summary of these analyses is presented in Table 1.

**Figure 5:**
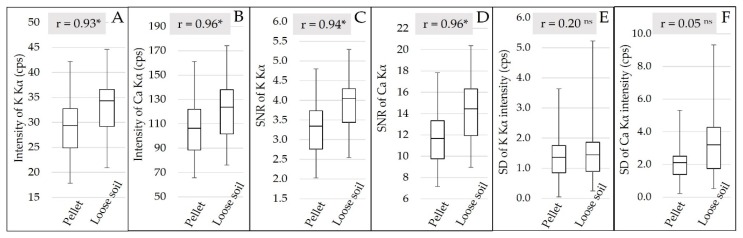
Box plot of the Kα emission line intensity of K and Ca (**A** and **B**, respectively), after averaging the replicates. Box plot of the signal-to-noise ratio (SNR) of the Kα emission lines of K and Ca (**C** and **D**, respectively), after averaging the replicates. Box plot of the standard deviation (SD) of the Kα emission line intensity of K and Ca (**E** and **F**, respectively) for the replicates. The Pearson correlation between the pellet and loose soil is also presented (correlations followed by * were significant at the probability level of 0.01; correlations followed by ^ns^ were not significant).

The qualitative evaluation of the XRF spectra (Figure 3) allowed us to identify the K and Ca emission lines, but no fluorescence emission was detected for Mg and P. The XRF intensity and the SNR of K and Ca were slightly higher for loose soil than pellet samples, and both had a highly significant correlation (r > 0.9) between pellet and loose soil, indicating that the changes promoted by sample preparation were well standardized for all samples (Figure 5). Despite the small gain in fluorescence intensity (an average of 4.21 and 15.31 cps for K and Ca, respectively) and in SNR (an average of 0.63 and 2.49 for K and Ca, respectively), when evaluating the behavior of the emission line intensity SD for the replicates, we can observe that the loose soil samples presented greater variation in relation to the pellets, both for K and Ca (Figure 5E,F, respectively). The replicate SD is an indicator of the reading precision. In this work, the lower precision of the loose soil samples might be related to their lower homogeneity in relation to the pelletized ones. Despite this loss of precision among the different replicates obtained in loose soil samples, the triplicate scans smoothed this effect. After averaging the replicates, the distribution of the XRF intensity showed a similar distribution between both sample preparations (Figure 5A,B).

**Figure 6:**
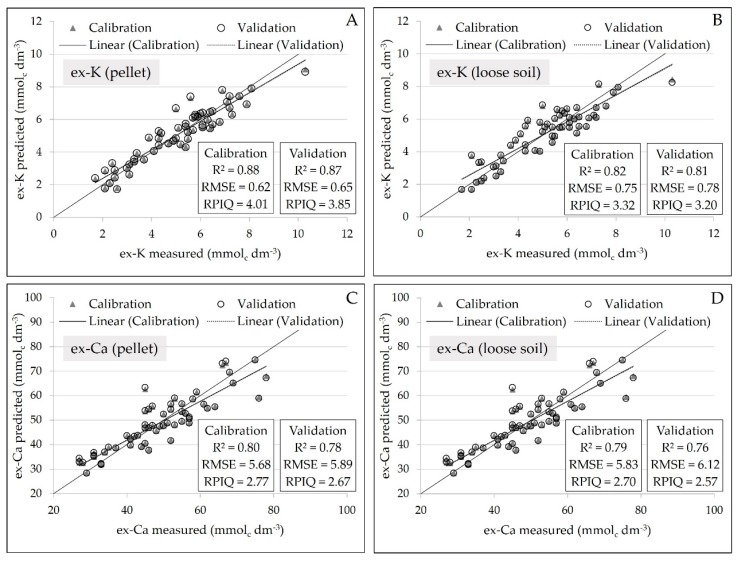
Scatter plots of measured versus predicted ex-K, for the pellets and loose soil (**A** and **B**, respectively), and of measured versus predicted ex-Ca, for the pellets and loose soil (**C** and **D**, respectively). Models were obtained using simple linear regressions with the Kα emission lines of each element (n = 58) and the validation was performed by “leave-one-out” full cross-validation.

Regarding the regression analysis, there was a slight reduction of precision in the calibration of ex-K and ex-Ca in loose powder soil samples, marked by a slight increase in error and reduction in R^2^ (Figure 6). Comparing the loose soil samples in relation to the pellet samples, the prediction error of ex-K increased from 0.65 to 0.78 mmol_c_ dm^−3^. Similarly, for the ex-Ca prediction, the error increased from 5.89 to 6.12 mmol_c_ dm^−3^. Moreover, concerning the R^2^ values, it oscillated from 0.87 to 0.81 for ex-K, and from 0.78 to 0.76 for ex-Ca. Besides that, all prediction models, obtained from both pellet and loose soil, showed excellent performance in their validation with RPIQ values above 2.5. 

## 4. Discussion

Although the XRF technique measures the total content of elements present in the soil, these sensors have been suggested as an auxiliary technique for evaluating fertility attributes [8,9,10]. In addition, interest in such equipment has recently increased due to its portability, enabling on-field studies [7] and in controlled environments such as hybrid laboratories [2,11]. Such applications are only compatible with minimal or no sample preparation.

Even if the XRF technique is flexible concerning sample preparation, there is a consensus that pellet preparation increases the data precision [15]. Pellet preparation consists of conforming and binding the samples into a specific shape. For pellet formation, the soil samples must be grinded to an extremely fine powder by using a grinder; furthermore, a proper binding agent can also be necessary [26]. In this work, a procedure for soil pellet preparation was evaluated, with and without binding agents. Sandy soils are more likely to not form pellets without the use of a binder [12]. In our work, it was perceived for samples with clay content around 175 g dm^−3^. During testing for optimizing binder concentration and ball milling time, it was observed that increasing the grinding time, as well as the binder concentration, improves the cohesion between the particles of the pellet. In contrast, higher concentrations of binder (e.g., 15% w w^−1^, in this work) make it difficult to homogenize it. It is known that the smaller the particle size to be pressurized is, the more resistant and cohesive the pellet will be [27]. However, pellet preparation in different soil sample sets can be optimized with different binder concentrations and milling time. To optimize these conditions, we suggest conducting preliminary tests, as described in this paper.

The reduction of the particle size, before pressing the material, promotes sample homogenization [26]. Both the element spatial patterns and the SD behavior on the replicates, clearly showed gain on homogeneity after pelletizing. The lower homogeneity of loose soil samples should be considered for determining the number of replicates during data acquisition with a pXRF device. Due to the high variability of element distribution in loose soil samples, a greater number of scans has to be acquired for more accurate representation of the sample surface, and then, different spectra can be averaged [12]. In this work, pXRF spectra were obtained in triplicate (at different positions) and were sufficient for a good characterization of both sample preparation samples.

One point to consider while milling samples is the possibility of contamination. Two types of contamination can occur, cross-contamination, due to inefficient cleaning when changing samples, and/or contamination with milling surfaces (e.g., agate and tungsten carbide), due to the abrasion between the sample and mill components. In addition to the careful execution of cleaning procedures, the milling surfaces should be considered in terms of hardness and elemental composition to avoid the risk of sample contamination [12]. In this work, the grinding in a ball mill made of tungsten carbide contaminated the samples with W. Soil samples usually have hard minerals, like silicates, and W should not be measured when tungsten carbide milling jars and balls are used for milling soil [28]. Specifically, in XRF analysis, W contamination can also be a problem for the determination of Si and Al, as described in the previous section. Iwansson and Landström [29] showed that this kind of contamination is higher in quartz-rich samples and increases with grinding time. When the element contamination (e.g., W) is the same one that is to be quantified, the values must be adjusted, discarded, or over-looked to avoid misinterpretation of the results [29].

Pelletizing also reduces surface roughness effects and increases the density of the material [26]. Theoretically, reducing sample roughness means decreasing the physical matrix effect, which would attenuate part of the fluorescence produced by the analytes, and increasing the density of samples and fluorescence intensity [14]. Thus, a higher fluorescence yield and an upsurge in the SNR were expected for the emission lines of the pelletized samples, due to the increase of their density and reduction of the physical matrix effect [13]. Nevertheless, this behavior was not observed in this work. In turn, the addition of binder (10% w w^−1^), as well as the contamination by W, and the differences in homogeneity found (Figure 4B,E), appear to be the factors that influenced this behavior, slightly altering the chemical composition of the pellet samples and, consequently, their fluorescence production.

Soil samples are naturally heterogeneous and therefore comminution procedures are generally recommended for improving matrix homogenization, and should yield homogeneous pellets [26]. Ultimately, this can avoid heterogeneity effects, such as grain size effect, mineralogical effect, and segregation, factors that cause errors in the XRF analysis [30]. However, in this work, the prediction models of ex-Ca and ex-K using pellets showed just a small performance gain over those obtained in loose powder soil samples. Using loose powder soil, prediction models for ex-Ca and ex-K calibrated with the 58 local samples obtained excellent performances, with RPIQ values over 2.5. These results are promising and encourage more detailed investigations on the application of the XRF technique. They even lead us to new questions such as: (i) how long will this calibration remain robust over different cropping seasons? (ii) is it possible to further reduce sample preparation without losing analytical quality? (iii) what would be the analytical performance in samples with field conditions (e.g., with different humidity and particle size patterns)? (iv) how to determine fields where the XRF sensor will have potential as an auxiliary tool alongside traditional fertility analysis methods?

Different works on temperate soils have shown good performance in predicting fertility attributes such as pH [9], cation exchange capacity (CEC) [10], base saturation (V%) [31], soil texture [32] and total content of different elements [8,11]. In Brazilian tropical soils, satisfactory performances have already been obtained for predictions of organic carbon and organic matter [33] and textural attributes [34]. Although, so far, the prediction of available nutrients has not been explored. The possibility of XRF application on soil samples that have just been dried and sieved (<2 mm), with satisfactory predictions of ex-K and ex-Ca using local models, is a promising alternative to increasing the efficiency of analytical procedures. This may intensify the amount of analysis in tropical soil samples without the need for wet chemistry methods. In addition, this level of sample preparation is compatible with evaluations using vis-NIR diffuse reflectance, opening the potential to exploit joint XRF and vis-NIR sensors on these types of samples. Moreover, the vis-NIR technique has great potential for obtaining information about texture, and organic and mineralogical components, which can synergistically complement the XRF information for a more complete characterization of the attributes of soil fertility [11].

A simple calibration method was applied in this work, using only the emission line of the elements of interest for predictive modeling. XRF spectra are multi-informational, allowing the measurement of several soil properties from a single scan. This is possible because each spectrum stores a large amount of information along with its emission lines and different types of scattering (e.g., Compton and Thomson scattering), not strictly related to the elementary constituents of the samples. An example is the prediction of organic carbon and organic matter in soil samples using the information contained in the scattering region of the spectra, as explored by Morona et al. [33]. In this sense, predictive modeling based on multivariate statistics and machine learning methods are an alternative to better exploit the hidden information present in XRF spectra [35], and it can enable robust determinations of fertility attributes that have an indirect relationship with inorganic soil constituents such as pH, CEC, V% and texture.

The evaluation of Mg and P using the XRF technique is challenging as they are light elements that produce fluorescence emission at low energy levels (between 1.2 and 2.2 keV) that are absorbed by atmospheric gases (N_2_, O_2_, and Ar) before reaching the detector. The maximum pseudo total content (Table 1) of P (670 mg kg^−1^) and Mg (790 mg kg^−1^) in our samples was not enough to produce X-ray fluorescence intensities detectable by the equipment. Therefore, even if there is a correlation between their pseudo total and available contents, direct calibrations for ex-P and ex-Mg (made with their own emission lines) have not been possible using the XRF spectra so far. However, this does not preclude an attempt of indirect calibrations, using other information present in the spectrum. Furthermore, the determination of these elements can be improved by using a vacuum system and changing the X-ray tube conditions to lower voltage (<10 keV) and increasing the current (10 to 15% of deadtime), which reduces air attenuation over Mg and P emission lines, and increases the fluorescence yield of these chemical elements. Some portable XRF equipment already allows the use of this condition and future work should be done to evaluate the use of a vacuum to improve the detection of light elements in soil samples to predict fertility attributes.

In addition, this study was conducted under a clayey lixisol, which is a representative and common type of soil in Brazilian tropical areas [36]. Therefore, this pioneering evaluation provided useful information to help XRF users—who aim to use this technique as a tool for practical soil analysis—to understand that the expected effects of sample preparation related to heterogeneity and physical matrix effects can be neglected. However, it is fundamental to also validate other types of soils with contrasting textural classes, which can proportionate distinct levels of physical matrix effects. 

## 5. Conclusions

The addition of a binder was decisive for improving the quality of the pellets. The best results for soil preparation in the form of pellets were obtained with samples prepared with cellulose binder at 10% w w^−1^ and ground for 20 min. 

Pressed pellets allowed a slight gain in performance over loose powder samples for the prediction of ex-K and ex-Ca. In spite of that, predictions in loose powder soil for ex-Ca and ex-K, calibrated with 58 local samples, obtained excellent performances in their validation, showing that it is possible to reduce the optimal sample preparation of XRF analyses for predicting soil nutrients. However, loose samples are less homogenous than pellets, and scanning loose soil samples in replicates is important for smoothing this effect.

The prediction models of ex-K and ex-Ca calibrated with local samples presented promising results. More detailed investigations are necessary to foster the application of the XRF technique in agricultural soil samples for determination of soil fertility attributes. Finally, XRF can serve as a complementary method to traditional laboratory analyses.

## Figures and Tables

**Table 1:** Descriptive statistics of exchangeable and pseudo total nutrients and the correlation between the respective nutrients.

Exchangeable Nutrients					Pseudo Total Contents			
	ex-P	ex-K	ex-Ca	ex-Mg	ptc P	ptc K	ptc Ca	ptc Mg
	mg dm^−3^	mmol_c_ dm^−3^			mg kg^−1^			
Min	7.00	1.70	27.00	11.00	405.31	154.04	492.42	411.59
Mean	20.20	5.14	49.12	26.28	489.93	318.10	750.51	607.38
Max	46.00	10.30	78.00	54.00	669.18	477.18	1225.91	789.65
SD	8.42	1.78	12.89	10.66	55.74	79.00	159.46	112.31
CV (%)	41.69	34.60	26.25	40.57	11.38	24.84	21.25	18.49
Correlation with pseudo total	0.79 *	0.67 *	0.83 *	0.52 *				

* Significant correlation at the probability level of 0.01.
